# Diabetes Knowledge, Behaviors, and Perceptions of Risk in Rural West Virginia Counties

**DOI:** 10.13023/jah.0303.05

**Published:** 2021-07-25

**Authors:** Ranjita Misra, Sara Farjo, Renee McGinnis, Megan Adelman Elavsky, Summer Kuhn, Catherine Morton-McSwain

**Affiliations:** West Virginia University; Cleveland Clinic Akron General Medical Center; West Virginia University

**Keywords:** Appalachia, diabetes risk factors, community screening

## Abstract

**Introduction:**

A little less than half of American adults have diabetes or pre-diabetes. In 2016, West Virginia (WV) had the highest percentage (15.2%) of adults with diagnosed diabetes in the U.S.

**Purpose:**

In partnership with the Health Sciences and Technology Academy (HSTA), a cross-sectional study was preformed to assess knowledge, behaviors, and perceptions of diabetes risk.

**Methods:**

Data was collected by trained HSTA students and teachers who lived in rural counties in WV. Information was assessed using validated surveys, and HbA1c was obtained by utilizing professional point-of-care (Bayer) kits.

**Results:**

Mean age and Body Mass Index (BMI) was 36.11±17.86 years and 27.80±6.09 kg/m^2^, respectively. More than half of the participants had a family history of diabetes (58.8%) and hypertension (60.2%), and a majority had elevated BMI (65.9%). However, only 29.2% rated their future risk for diabetes as moderate to high. Eighty percent (80%) had an inadequate amount of weekly exercise, and 36% had lower quality of diet. Overall, dietary quality and diabetes knowledge was associated with a low to moderate diabetes risk score; risk score positively correlated with higher HbA1c (r=0.439, P<.001). Participants’ HbA1c, perceived future risk of diabetes and family history of diabetes emerged as significant predictors of diabetes risk in the regression model, controlling for health behavior and diabetes knowledge.

**Implications:**

HbA1c, perceived future risk of diabetes and family history of diabetes may be the best predictors of developing diabetes in the future and, therefore, are important to assess during community screening. Perception of diabetes risk is lower than actual diabetes risk in WV.

## INTRODUCTION

Diabetes is a major public health concern as it continues to be a serious, highly prevalent chronic health condition, and in the U.S. accounts for the seventh leading cause of death.^1^ Overall, 13% and 34.5% of the United States adult population are estimated to have diabetes and prediabetes respectively.^1^ Diabetes is associated with many micro and macrovascular complications such as cardiovascular disease, nephropathy, and retinopathy which leads to a high health and economic burden. Complication rates from diabetes have increased among young and middle-aged adults.^2^ From 2012 to 2017, the estimated healthcare costs associated with diabetes increased from 261 billion to 327 billion dollars.^1^

Individuals with prediabetes are at increased risk for developing diabetes.^2^ Unfortunately, it is estimated only 15.3% of the 88 million adults who have prediabetes have been formally diagnosed and are aware of the diagnosis.^2^ In 2015, the U.S. Preventive Services Task Force recommended all adults aged 40–70 years who are overweight or obese be screened for abnormal glucose levels as a part of a cardiovascular risk assessment.^3^

West Virginians are disproportionately burdened by diabetes. West Virginia (WV) had the highest percentage (15.2%) of adults with diagnosed diabetes out of all U.S. territories and states in 2016.^3^ Yet, the prevalence of prediabetes is ranked twelfth at 8.5%.^2^ A diabetes risk survey conducted throughout 12 rural counties in WV showed that 61.8% of participants were at high risk for prediabetes.^4^,^5^ This contrast emphasizes the screening gaps in West Virginia.

Diabetes screening can help identify high-risk individuals, yet, there are multiple screening barriers to successful care including access, distance, and transportation, particularly in rural (WV is 3rd in the nation), medically underserved (91% of counties), and impoverished communities in this state.^6^ The few studies on community outreach for diabetes screening in rural WV demonstrates that the coupling of community organizations with healthcare systems helps improve continuum of care and patient-centric outcomes. Communities can utilize unique populations—such as early learners—to help with this bridge.^1^ In the current study, the goal was to evaluate diabetes risk throughout eight counties in WV by utilizing local high school students from the Health Sciences and Technology Academy (HSTA) at West Virginia University (WVU) to perform in-person community screenings. The screenings involved a survey assessing health history, behaviors, knowledge, and perception of diabetes. In addition, anthropometric data was obtained on all participants, and rapid HbA1c tests were performed. This information will help inform future studies to assess gaps in care, potential populations to target for community outreach, and the possibility of utilizing early learners (i.e., high school students) for successful screenings. In further identifying the specific factors most associated with developing diabetes in WV, researchers would be better equipped to identify those most at risk and over time reduce disease burden.

## METHODS

Diabetes screening was conducted in multiple counties to assess diabetes risk using academic partners, HSTA, and community leaders. The screenings were completed by HSTA teachers, staff, and students. The HSTA personnel were trained for one week at the WVU Health Science Center by a multidisciplinary team of researchers and practitioners from Medicine, Pharmacy, Public Health, and Physical Therapy. The topics included diabetes pathophysiology; disease risk factors; lifestyle modifications; anthropometrics; ethics protocol; HbA1c testing; and the process of obtaining consent surveys. Historically, HSTA personnel worked with community leaders in various counties and provided a range of research and educational projects. Therefore, utilizing the HSTA network to test the effectiveness of diabetes screening and referrals in WV communities is a sustainable long-term model in WV. The project was approved by the WVU IRB board prior to data collection in Spring 2019.

Data collection occurred in McDowell, Cabell, Lincoln, Monongalia, Marion, Kanawha, Braxton, and Webster counties. Chosen counties were primarily selected based on the location of HSTA students and faculty and the pre-established relationships with local organizations (i.e., standing community fairs, churches). Demographics of participants were mostly similar from all the counties surveyed for this study. Given the familiarity of HSTA with community organizations and leaders, several screenings were offered to maximize participation. Use of HSTA students and alumni provided opportunities to maximize recruitment of interested participants, who are generally from rural and minority communities. Participants were enrolled based on participant interest and advertising and outreach at the community events. Upon providing consent, participants completed the 4-page survey and the HbA1c test. The survey included demographics, health behaviors, diabetes knowledge and perceptions of future risk. Eighty-three participants were screened in eight rural counties (21 ZIP codes). Participants included adults 18 years and older since those less than 18 years are unlikely to have higher risk for diabetes. Data were collected by trained HSTA research personnel. The primary outcome was diabetes risk as assessed by the investigational survey and rapid HbA1c point-of-care test.

### Diabetes Risk Assessment

A combination of surveys and rapid HbA1c point-of-care tests were used to predict the risk for prediabetes or undiagnosed diabetes.^7^ This approach involved the following two steps:

1. completion of the Centers for Disease Control and Prevention (CDC)’s prediabetes risk test.^8^ The risk factors included older age, gender, elevated weight based on height, physical inactivity, family history of diabetes, and gestational diabetes or delivery of a large baby. A patient was defined as “high-risk” for diabetes if his/her diabetes risk score was ≥5 based on this assessment. The tool was cross-validated using NHANES III data.^9^
2. a drop of blood obtained by finger stick to analyze HbA1c. The point-of-care A1C Now monitoring kit by Bayer was utilized as it has shown to be accurate and correlates well with standardized laboratory testing.^10^ The use of HbA1c was preferred over glucose level because a finger prick can easily be collected at any time during the day by research personnel. It also estimates average blood glucose levels over the past 3 months. The A1C Now is approved by the Food and Drug Administration for monitoring of HbA1c and is available for over the counter or professional use.^11^,^12^

Participants with no known history of diabetes were able to be identified due to self-reported medical history. HbA1c levels of >5.7% were considered high-risk individuals with impaired glucose tolerance or possibly undiagnosed prediabetes or diabetes; referral to a medical provider was recommended. Individuals were provided their diabetes risk score; handouts on how to interpret their HbA1c score, blood pressure, percent body fat, and waist circumference; and established provider contact information in their respective communities. Furthermore, high-risk individuals were encouraged to follow-up with a healthcare provider for repeat testing and confirmation as per the American Diabetes Association (ADA) recommendations.^13^

Perceptions of future diabetes risk was assessed by a question “how would you rate your risk of developing diabetes in the future? (1=No risk to 5=Extremely high risk).^14^

Family history of diabetes is an important risk factor for developing diabetes in the future. Hence, participants were asked whether they had a family history of diabetes among grandparents, uncle, aunt, parents, or siblings. The response was coded as 1=Yes, 0=No.^14^

### Healthy Lifestyle Habits

Healthy lifestyle behaviors including tobacco use, dietary habits, and physical activity were assessed. The Rapid Eating Assessment for Participants (shortened version; REAP-S) was used to assess participants’ diet quality.^15^ Items were summed with scores ranging from 13 to 39; higher score indicated a higher diet quality. Cronbach’s alpha (α) of the REAP-S was 0.74, indicating good reliability. The following yes or no questions were assessed in the survey: “do you read food labels”, “do you smoke”, and “are you physically active”. Additionally, to assess activity level, the survey questioned “how often do you exercise for periods of at least 30 minutes?”; option responses included once/week, 1–2 times, 3–4 times, and 5 or more times/week.

#### Diabetes Knowledge

The diabetes knowledge questions were adapted from a prior survey developed by the first author and two medical/public health professional experts.^16^ Knowledge was assessed by 21 questions that focused on general knowledge of type 2 diabetes, risk factors, symptoms, diagnosis, treatment, and prevention. A scoring system was developed where each correct response received one point, and an incorrect/no response received zero. Questions with multiple responses (e.g., knowledge of symptoms and prevention approaches) were coded similarly. The questions were summed for a total composite diabetes knowledge score (range 0–21) with a higher score indicating higher knowledge of diabetes. Cronbach’s alpha (α) of the diabetes knowledge scale was 0.785, indicating good reliability.

#### Demographic Information

In addition, participants’ demographic characteristics (age, gender, educational level, and self-reported height and weight for a calculated BMI) were collected.

The Statistical Program for Social Sciences (SPSS) system (version 26.0) was utilized. Basic descriptive and inferential statistics were completed for demographic, health behavior, diabetes knowledge, perceptions, and risk factors. Analysis of variance was used to evaluate the difference in study variables by participants in the low- and high-diabetes risk categories. Linear regression examined the association of diabetes risk score (dependent variable) with predictor variables [HbA1c, knowledge, family history of diabetes, health behaviors (diet quality, read food label and exercise), and perception of developing diabetes in the future].

For multivariate analysis, predictor variables were limited to 7 predictors in a model considering the sample size was 83 participants and a minimum of 10 samples for each predictor variable recommended in multivariate regression models.^17^ Participants who reported having diagnosed diabetes (n=8; 9.6%) were removed from the regression analysis. While unstandardized coefficients provided information on the effect of each predictor variable on diabetes risk (bivariate), standardized (beta) coefficients were used to interpret the strength of the effect of each predictor variable on diabetes risk controlling for other variables in the model. Model significance and adjusted R^2^ described the significance of the linear regression model as well as percent variance explained by the significant predictors for participant’s diabetes risk score. P <0.05 was used as acceptance level for statistical significance.

## RESULTS

The sample consisted of 83 individuals from eight rural counties. The majority were female (67.5%), non-Hispanic whites (82.9%), with a mean age of 36.11±17.86 years (range 18 to 75; Table 1). Participants were often classified as overweight (average BMI: 27.88±6.09 kg/m^2^), with an average percentage body fat of 30.1%±14.87. In the three top-populated counties representing the sample (n=68), 39 (57.4%) had a college degree. Average HbA1c was 4.97% ± 0.59. Most patients (90.4%) had no prior diagnosis of diabetes mellitus. Five participants (7.2%) who did not have a prior diagnosis of diabetes had HbA1c values indicative of prediabetes or diabetes. Overall, participants were educated, with 61% reporting they had some college or a college degree. Most participants had a family history of diabetes (58.8%), did not have a personal history of hypertension (90.2%), and reported they had a healthcare provider (84.8%).

A large majority of the participants did not use any form of tobacco (88.6%). Although 59.5% indicated that they were physically active, less than one-fifth (20.8%) met the Surgeon General’s recommended level of exercise duration of at least 30 minutes for 5 or more days per week.

Overall, dietary quality assessed from the REAP-S was low to moderate 36% scored between 16 and 27, indicating lower diet quality, 58% scored from 28 to 34, and 6% scored 35–37 indicating a higher diet quality. Furthermore, only a little more than one-third of the participants (39.8%) read food labels to identify nutrients in packaged foods. Further exploration indicated individuals sought to use information on calories (67.5%), carbohydrate (62.3%) and/or sugar (71.4%) when they read food labels.

Participants self-rated their physical health as mostly good (36.1%) or very good (30.1%). Twelve percent rated as excellent, with 2.4% and 19.3% rating it as poor and fair, respectively. However, looking at the health behavior of participants who rated their health as good to excellent category,76.9% lacked regular physical activity (30 min per day for 5 or more days a week) and 60% were overweight/obese. In addition, 33% and 46.2% of these respondents self-reported a family history of diabetes and/or hypertension.

The mean diabetes knowledge score of the participants was 13.13 (SD 4.99; range 0–21), indicating a low to moderate level of knowledge with a third of participants unaware of diabetes implications and its associated risk factors and complications. Approximately 70% of participants perceived no to low risk of developing diabetes in the future, and only 29.2% rated their risk as moderate to high. Participants with a family history of diabetes were significantly more likely to perceive a future risk of diabetes than those with no family history (p=0.001).

### Diabetes Risk and Perceptions

Mean diabetes risk score, computed from the CDC 7-item questions, was 3.19 (SD 1.59; range 0–11). Only one-fifth (19.3%) of individuals were considered at high-risk based on the diabetes risk score. The two most prevalent risk factors for diabetes were elevated BMI and a family history of diabetes.

A comparison of the participant characteristics between the low risk versus high-risk group are shown in Table 2. Significant differences in demographic characteristics were noted between the two groups by age, BMI, family history of diabetes, and health behaviors. For example, high-risk individuals were more likely to be older than low-risk participants (p<0.001). In addition, they were overweight or obese (p=0.03), had a family history of diabetes (p<0.001) and a diagnosis of hypertension (p=0.013). Individuals who met the high-risk criteria had significantly higher HbA1c levels as compared to the low-risk group (p=0.002; Table 2). Female high-risk participants also reported at least one baby who weighed more than 9 pounds at birth during their pregnancy (p=0.02). However, no difference was noted in diabetes knowledge and diet quality score between the two groups (p>0.005).

Bivariate associations (not shown in Table) showed significant positive bivariate association between diabetes risk score and HbA1c levels (r=0.439, p≤0.001). Diabetes risk score was also positively associated with diabetes knowledge (r=0.25, p=0.023) and perception of developing diabetes in the future (r=0.431, p=<0.001), as well as known risk variables including BMI, percentage body fat, age, family history of diabetes, and hypertension.

Multivariate regression model examined the significant factors which contribute to participant’s diabetes risk score (Table 3). Results showed the following variables were significant predictors of diabetes risk score in the model: higher HbA1c (standardized beta = 0.364; p =0.001), perceived future risk (standardized beta = 0.248; p = 0.022) and having a family history of diabetes (standardized beta = 0.435; p <0.001). The model was significant and explained 47.5% of the variance (F=8.62; p<0.002; Adjusted R2 =0.475). Reading food labels and having a higher diet quality approached statistical significance (p = 0.09 and 0.07, respectively) in the regression model. A positive association (standardized beta) between diabetes risk score and the three predictor variables indicated that participants with higher HbA1c, a family history of diabetes and higher perceived diabetes risk had higher diabetes scores. In addition, family history of diabetes had the strongest effect on participant’s diabetes risk score followed by glycosylated hemoglobin level and future risk perception.

## IMPLICATIONS

Diabetes is a health burden with roughly 29.1 million Americans currently diagnosed, with a disproportionate amount affected in WV.^18^ While approximately 8.5% of West Virginians have been formally diagnosed with prediabetes, there is tremendous misalignment between this rate and the estimated national average (34.5%), which highlights a large gap in care and screening.^1^,^2^ This is particularly concerning as prediabetes puts individuals at higher risk of developing diabetes; studies indicate ~5%–30% of patients with prediabetes are later diagnosed with diabetes.^19^ Aggressive intervention—such as weight loss and increased exercise—can drastically reduce further progression.^20^

Surprisingly, a low percentage of the participants (19.3%) were deemed to be high-risk compared to prior studies.^21^ This low percentage is inconsistent with current community estimated rates of prediabetes and diabetes in WV. This highlights potential selection bias from the participants who were younger (average age <40 years) and had an overweight but not obese BMI (average: 27 kg/m^2^). Additionally, a large majority had established primary care providers. Therefore, most of the individuals who participated in the community outreach events did not require further healthcare intervention or education. However, this study highlighted potential gaps in care with 7.24% patients having HbA1c results consistent with prediabetes or diabetes.

Prior studies demonstrate conflicting results of the impact of community engagement programs.^22^,^23^ The American Association of Diabetes Educators’ White Paper from 2014 recommended community-based screening be organized to target known areas and groups where individuals are at higher risk or tend to have undiagnosed cases (e.g., among individuals “over 45 years of age, ethnic and racial minorities, and women with gestational diabetes”).^24^ In highlighting the results discussed above, it is important to note that HSTA network has successfully worked with community leaders to increase the likelihood of the community members participating in research projects. It is critical that teams fully research area populations and communities prior to organizing events for maximum impact and outreach efficacy. Preference should be placed on location with low healthcare resources, lower educational and/or socioeconomic population, and/or rural populations. For individuals hoping to start community outreach events, it is critical to establish buy-in in the community. This can be done through community leaders and partnerships. A community assessment may be beneficial to identify areas of opportunity and select community champions.

Diabetes knowledge and risk have previously found to correlate with HbA1c levels, with those individuals demonstrating knowledge of diabetes risk having lower HbA1c levels.^25^ Interestingly, this study demonstrated increased diabetes risk was associated with a higher level of diabetes knowledge. This may have been impacted by the number of patients who had a family member with diabetes, and therefore, a first-hand knowledge of diabetes implications. Overall, this population had a good baseline knowledge of diabetes, its associated complications, and risk factors; a majority (70%) perceived they had little or no risk of later developing diabetes in the future which aligned with the current low diabetes risk score. However, there were some misalignments in some aspects of the self-scoring and objective data; this may be explained by patients’ poor perception of self-health, as previously identified in the literature.^26^ An example of this within this study population is a high percentage rating themselves as “being physically active” while a minority reach the recommended minutes exercised per week. Further, perception of diabetes risk was lower than actual diabetes risk in rural WV.

Additionally, the community outreach provided an opportunity to compare objective (HbA1c) to subjective (survey results) data. Participants’ HbA1c, perception of future risk of diabetes, and family history of diabetes emerged as significant predictors of diabetes risk score, controlling for health behavior and diabetes knowledge. As the diabetes screening tool has previously been validated, these results align with prior evaluations. While quality diet was not found to be a predictor of diabetes risk score but approached significance, this population survey highlighted a significant area of need as majority of participants self-evaluated their diet quality as lower-to-moderate. Research shows that perceived diet quality in American adults is associated with actual diet quality.^27^ The rural and geographical isolation and food deserts limit access to high quality and low-cost food items. Gaining consistent access to affordable options can be a struggle in the rural community, so education should be focused on affordability and unique strategies to meet these needs.^28^ Overall, these interventions and education could help to increase high-quality foods, which is associated with a lower risk of diabetes especially among those with a high BMI.^29^

This study had several limitations including the sample size of the population, lack of repeat data and follow up, and limited objective measurements. Generalizability of the results to the WV population is limited due to the small sample size and self-reported data on health behavior and risk perceptions. Additionally, we recognize there may have been selection bias as Kanawha county is one of the largest counties in the state with a highly affluent area; the participants were more educated and included a higher percentage of minorities than the general WV population. While students would advertise for the community outreach, participants may have already been engaged and interested in their own healthcare which may have biased results. The community outreach event locations were primarily limited to where HSTA representatives were located and not based on areas where healthcare resources are lacking, which would have been preferable.

Ideally for community-based screenings, all patients who complete surveys should have objective lab screening. However, in those who were screened, patients could only be told they were “high-risk” and confirmatory diagnosis testing would be needed as per the ADA guidelines. Hence, HSTA personnel provided respondents with a list of local providers, though it is unknown if the patients had further engagement with the healthcare system. Ideally, coordinated community-based screenings with a primary care office or clinics that accept patients with or without health insurance who warrant further follow up will allow for a better transition of care.

Public health screening programs have historically helped control health epidemics and are a vital tool to help manage chronic disease awareness and management. In providing additional resources to communities, outreach events —such as those highlighted in this writing—illustrate the benefits of early identification and appropriate referral to the healthcare system. Similarly, these are ideal avenues to promote community wellness, impacting population health management. While this research focused on metabolic and diabetes screening, policymakers should consider the benefits of community needs assessments and how partnership with community organizations and the healthcare system can beneficially support a community.

The current study advances research in diabetes screening by examining participants’ knowledge, behaviors, and perceptions of risk of developing diabetes while screening for prediabetes and diabetes. While further steps will need to be researched and optimized, we believe an emphasis on using trusted local networks will benefit rural communities for community engagement outreach and education.

**SUMMARY BOX**

**What is already known about this topic?** Diabetes is a major health problem in the Appalachian region, and WV, an entirely Appalachian state, has the highest percentage of diagnosed diabetes in the nation.

**What is added by this report?** This report addresses diabetes knowledge, behaviors, and perceptions of future diabetes risk within rural WV during community screening to gain a better understanding of why diabetes is so prevalent and how diabetes risk can be better identified.

**What are the implications for future research?** This research has illustrated there is overall good baseline diabetes knowledge, inadequate physical activity and diet quality, and a lower perception of diabetes risk when compared to actual risk in WV. HbA1c, perceived future risk of diabetes and family history of diabetes may also be the best predictors of developing diabetes.

## Figures and Tables

**Table 1:** Sample Characteristics of Participants

Variables	Total (n=83)		High Diabetes Risk^a^ (n=14; 49.8%)		Low Diabetes Risk (n=69; 34.2%)		
	Freq	%	Freq	%	Freq	%	P-value
**Gender**							0.531
Female	56	67.5	11	68.8	42	65.6	
Male	27	32.5	5	31.3	22	34.4	
**Ethnicity**							0.319
Non-Hispanic whites	68	82.9	15	93.8	51	81.0	
Minorities	14	17.1	1	6.3	12	19.0	
**Age (years)**	Mean = 36.11 ± 17.86 years						**<0.001**
18–44	54	67.5	2	12.5	49	80.3	
45–64	19	23.8	9	56.3	10	16.4	
≥ 65	7	8.8	5	31.3	2	3.3	
**Education**							0.114
≤ High school grad	32	39.0	3	20.0	26	40.6	
College grad Or Some College	50	61.0	12	80.0	38	59.4	
**Exercise 30 min**							**0.046**
< once a week	15	19.5	6	42.9	9	14.5	
1–2 times per week	29	37.7	2	14.3	26	41.9	
3–4 or more times/week	17	22.1	2	14.3	15	24.2	
5 or more times/week	16	20.8	4	28.5	12	19.4	
**Body Mass Index (category)**	Mean = 27.88 ± 6.09						**0.031**
Under/normal	29	35.8	1	6.3	25	40.3	
Overweight	25	30.9	8	50.0	17	27.4	
Obese	27	33.3	7	43.8	20	32.3	
**Read Food Labels**							***0.023***
Always/Often	33	39.8	9	43.8	23	35.9	
Sometimes/Never	50	60.2	7	56.4	41	64.1	
**Tobacco Use**							0.432
Yes	9	11.4	1	6.7	8	13.1	
No	70	88.6	14	93.3	53	86.9	
**Personal Hx of Hypertension**							**0.038**
Yes	8	9.8	4	26.7	4	6.3	
No	74	90.2	11	73.3	60	93.8	
**Family Hx of Hypertension**							**0.032**
Yes	50	60.2	12	80.0	32	50.0	
No	33	39.8	3	20.0	32	50.0	
**Family Hx of Diabetes** b							
Yes	47	58.8	16	100	17	26.6	**<0.001**
No	33	41.3	0	0	47	73.4	

Note: HCP = Healthcare provider; Hx=history; P-values: + < 0.10, * < 0.05, ** < 0.01

a Diabetes risk was assessed by the Centers for Disease Control and Prevention (CDC)’s prediabetes risk test. High risk for diabetes was defined as diabetes risk score ≥ 5.

b Family history of diabetes included the disease among their grandparents, uncle, aunt, parents, or siblings. The response was coded as 1=Yes, 0=No.

Total number of participants may not add up 100% due to missing responses.

**Table 2:** Diabetes Knowledge and HbA1c by Participant’s Diabetes Risk

Diabetes Risk Factors	Low Risk Score (n=64)^c^		High Risk Score (n=16)^c^		
	Freq	%	Freq	%	P-value
**Gender**					0.579
Male (1 point)	21	26.3	5	6.3	
Female (0 point)	43	53.8	11	13.8	
**Parent, sister or brother with diabetes**					**<0.001**
No (0 points)	47	58.7	0	0	
Yes (1 point)	17	21.3	16	20.0	
**Age**					**<0.001**
< 40 years (0 points)	50	62.5	3	3.8	
40–49 years (1 point)	2	2.5	0	0	
50 years (2 points)	12	15.0	13	16.3	
**Diagnosed with high blood pressure**					**0.013**
No (0 point)	62	77.5	12	15.0	
Yes (1 point)	2	2.5	4	5.0	
**Weight is more than listed for height**					**0.011**
No (0 point)	24	30.4	1	1.3	
Yes (1–3 points)	39	49.4	15	19.0	
**Physically active**					0.612
No (0 point)	17	21.5	4	5.1	
Yes (1 point)	47	59.5	11	13.9	
**Baby weigh more than 9 pounds at birth** *					**0.025**
No (0 point)	41	77.4	8	15.1	
Yes (1 point)	1	1.9	3	5.7	
	**Mean**	**SD**	**Mean**	**SD**	
**Diabetes Risk Score**	2.75	1.02	5.56	0.81	**<0.001**
**Diabetes Knowledge** a	12.75	5.35	14.81	3.44	0.147
**Diet Quality** b	28.10	4.82	30.37	3.59	0.083
**HbA1c (%)**	4.97	0.59	5.54	0.61	**0.002**
**BMI (kg/m** **^2^** **)**	27.29	5.99	30.85	5.74	0.03
**Percent Body Fat (%)**	28.84	15.6	37.38	11.34	0.13
**Waist Circumference (in)**	36.12	6.06	39.46	6.24	0.07

Note: Diabetes risk was assessed by the Centers for Disease Control and Prevention’s prediabetes risk test. High-risk for pre-diabetes/diabetes is defined by risk score ≥5

* Only females were selected for this analysis

a Diabetes knowledge assessed general knowledge of type 2 diabetes, risk factors, symptoms, diagnosis, treatment, and prevention. A higher score represents higher knowledge.

b Diet Quality was assessed by the Rapid Eating Assessment for Participants [REAP-S]. A higher score indicated a higher diet quality.

c Total number of participants may not add up 100% due to missing responses.

**Table 3:** Association of Diabetes Risk Score with Diabetes Knowledge, Future Risk, Health Behaviors, and HbA1c

Predictor Variables	Unstandardized B	Standardized Beta	P-Value
HbA1c	0.933	0.364	**0.001** **
Read Food Label	−0.513	−0.179	0.092+
Diet Quality^a^	0.054	0.188	0.074+
Exercise	−0.015	−0.012	0.912
Diabetes Knowledge^b^	0.008	0.028	0.778
Perception of Future Diabetes Risk^c^	0.439	0.248	**0.022** *
Family History of Diabetes^d^	1.298	0.435	**<0.001** **

Note: P-values:

+ < 0.10,

* < 0.05,

** < 0.01

Dependent variable, diabetes risk score, was assessed by the Centers for Disease Control and Prevention (CDC)’s prediabetes risk test. A higher score represents higher risk.

a Diet Quality was assessed by the Rapid Eating Assessment for Participants [REAP-S]. The items (range 13–39) were summed with higher score indicated a higher diet quality.

b Diabetes knowledge was assessed by 21 questions that focused on general knowledge of type 2 diabetes, risk factors, symptoms, diagnosis, treatment, and prevention. A higher score represents higher knowledge.

c Perception of future diabetes risk was assessed by a question “how would you rate your risk of developing diabetes in the future? (1=No risk to 5=Extremely high risk).

d Family history of diabetes included the disease among their grandparents, uncle, aunt, parents, or siblings. The response was coded as 1=Yes, 0=No
